# N-glycan profiling of tissue samples to aid breast cancer subtyping

**DOI:** 10.1038/s41598-023-51021-3

**Published:** 2024-01-03

**Authors:** Iva Benesova, Rudolf Nenutil, Adam Urminsky, Erika Lattova, Lukas Uhrik, Peter Grell, Filip Zavadil Kokas, Jana Halamkova, Zbynek Zdrahal, Borivoj Vojtesek, Milos V. Novotny, Lenka Hernychova

**Affiliations:** 1 Research Centre for Applied Molecular Oncology, Masaryk Memorial Cancer Institute, Zluty kopec 7, 656 53 Brno, Czech Republic; 2 Department of Pathology, Masaryk Memorial Cancer Institute, Zluty kopec 7, 656 53 Brno, Czech Republic; 3 National Center for Biomolecular Research, Faculty of Science, Masaryk University, Kotlarska 2, Brno, Czech Republic; 4 Central European Institute of Technology, Masaryk University, Kamenice 753/5, 625 00 Brno, Czech Republic; 5 Department of Comprehensive Cancer Care, Masaryk Memorial Cancer Institute, Zluty kopec 7, 656 53 Brno, Czech Republic; 6 Department of Chemistry, Indiana University, 800 E. Kirkwood Avenue, Bloomington, IN 47405 USA

**Keywords:** Biochemistry, Cancer, Biomarkers, Medical research, Oncology

## Abstract

Breast cancer is a highly heterogeneous disease. Its intrinsic subtype classification for diagnosis and choice of therapy traditionally relies on the presence of characteristic receptors. Unfortunately, this classification is often not sufficient for precise prediction of disease prognosis and treatment efficacy. The N-glycan profiles of 145 tumors and 10 healthy breast tissues were determined using Matrix-Assisted Laser Desorption-Ionization Time-of-Flight Mass Spectrometry. The tumor samples were classified into Mucinous, Lobular, No-Special-Type, Human Epidermal Growth Factor 2 + , and Triple-Negative Breast Cancer subtypes. Statistical analysis was conducted using the reproducibility-optimized test statistic software package in R, and the Wilcoxon rank sum test with continuity correction. In total, 92 N-glycans were detected and quantified, with 59 consistently observed in over half of the samples. Significant variations in N-glycan signals were found among subtypes. Mucinous tumor samples exhibited the most distinct changes, with 28 significantly altered N-glycan signals. Increased levels of tri- and tetra-antennary N-glycans were notably present in this subtype. Triple-Negative Breast Cancer showed more N-glycans with additional mannose units, a factor associated with cancer progression. Individual N-glycans differentiated Human Epidermal Growth Factor 2 + , No-Special-Type, and Lobular cancers, whereas lower fucosylation and branching levels were found in N-glycans significantly increased in Luminal subtypes (Lobular and No-Special-Type tumors). Clinically normal breast tissues featured a higher abundance of signals corresponding to N-glycans with bisecting moiety. This research confirms that histologically distinct breast cancer subtypes have a quantitatively unique set of N-glycans linked to clinical parameters like tumor size, proliferative rate, lymphovascular invasion, and metastases to lymph nodes. The presented results provide novel information that N-glycan profiling could accurately classify human breast cancer samples, offer stratification of patients, and ongoing disease monitoring.

## Introduction

Breast cancer is the most common cancer in women worldwide, with around 2.3 million new cases diagnosed in 2020^1,2^. Screening and early diagnosis rely on imaging methods such as digital mammography, sonography, or magnetic resonance imaging^3^. Breast cancer is heterogeneous in its clinical, morphological, and molecular attributes. This heterogeneity cannot be readily described by parameters such as tumor size, lymph node involvement, histological type, and grade. Serum biomarkers such as Cancer Antigen 15-3 (CA 15-3) or Carcinoembryonic Antigen (CEA) are applied mainly for monitoring treatment response in patients with advanced disease^4^ and do not provide adequate answers in terms of early diagnosis, prognosis or subtyping of breast cancer. Breast cancer subtypes can be classified based on immunohistochemical (IHC) determination of key proteins, gene expression profiles, or histology. The main IHC markers are Estrogen Receptor (ER), Progesterone Receptor (PR), Human Epidermal Growth Factor 2 (HER2), and the proliferation marker Ki67. The most common (around 70–80% in the Caucasian population) **Luminal** (L) cancers can be further subdivided. **Luminal A** (LA) which is the most common subtype, representing 50–60% of breast cancers is characterized by the presence of ER, usually high PR, absent or low HER2 (without gene amplification), and low rate of proliferation^5^. **Luminal B** (LB) tumors represent between 10 and 20% of all breast cancers are often more aggressive than LA tumors, and have a worse prognosis. LB cancers are ER-positive, usually with low or absent PR, and high Ki67 proliferation. The **HER2** subtype corresponds to approximately 10% of breast cancers and is characterized by high HER2 levels due to gene amplification. Overexpression of HER2 promotes the growth of cancer cells these tumors show a less favorable prognosis than HER2- negative tumors. Targeted HER2 antibody therapies, such as trastuzumab, are successfully used for treatment to block cell growth. **Triple-negative breast cancer** (TNBC) covers 10–20% of all breast carcinomas and represents a heterogeneous category, defined by the absence of the three key breast cancer receptors: ER, PR, and HER2. TNBC is a more aggressive type of tumor with a faster growth rate and a higher risk of metastasis. It has fewer treatment options and tends to have a worse prognosis. These tumors may be further divided into several subgroups^6,7^ partially defined by expression of Epidermal Growth Factor Receptor (EGFR), cytokeratin 5/6, p40, (ΔNp63), and Androgen Receptor (AR), but these features are overlapping.

The histological classification of breast cancer^8^ does not reflect intrinsic subtypes or expression profiles. It is predominantly morphology-based and divides cancers into (i) No-specified-type (NST, the most frequent group), (ii) Special types (most frequent are lobular, mucinous, and tubular), and (iii) Rare types. Other approaches to subtyping are based on gene expression-based platforms best-known are Oncotype DX®^9^, MammaPrint®^10^, or EndoPredict^11^. Both IHC and gene expression approaches are used to identify patients who are likely to benefit from hormonal or cytotoxic therapy. Consequently, there is a pressing need to identify and validate additional molecular markers for this disease to reliably differentiate its subtypes and facilitate better patient outcomes. With the recent development of “omic” technologies, including glycomics and glycoproteomics, such methodologies are being proposed for the detection and classification of breast cancer, as well as prognosis and prediction of therapeutic outcomes^12^. Glycoproteins are gradually becoming commonly used biomarkers associated with a variety of diseases^13^. Whilst unusual types of glycosylation in cancer were identified decades ago^14,15^, further investigations have provided structural information and data on the levels of glycan variants in cancer using a variety of sample types, including tissue, serum, and other body fluids^16–20^.

Glycomic mass spectrometry (MS) studies relating to breast cancer date back a decade^21–23^, demonstrating glycan profiles ranging from high-mannose structures to tetra-antennary glycans with a high degree of sialylation. This comprehensive coverage of glycan profiles has been facilitated through the use of permethylation derivatization. The permethylation procedure^24^ stabilizes sialic acid residues and prevents the migration of fucose residues that are otherwise labile during MS measurements^25^. This methodology involves Matrix-Assisted Laser Desorption-Ionization Time-of-Flight (MALDI-TOF) MS in glycan profiling, which can be automated and multiplexed^26^. The reported glycomic analyses of breast cancer tissues are considerably less frequent than those of serum and plasma. At least in part, the difficulties with reproducible processing of tissue samples may also be responsible for this situation.

Toward the ultimate goal of identifying changes in glycosylation associated with distinct types of breast cancer, whether tissue-originated or epiphenomenal, we have collected specimens from 150 patients and identified the glycomic profiles associated with each cohort. The study further deals with statistical analysis of the data to better characterize intrinsic and unclassified types of mammary tumors, to be subsequently correlated with serum glycomic analyses of the same patients. Detailed clinical descriptions of the patient cohort allowed us to pinpoint stage-specific changes in overall sialylation and fucosylation, in addition to assessing changes in the abundance of oligomannose N-glycans. Herein, glycan permethylation combined with MALDI-TOF–MS enabled a comprehensive and quantitative representation of structures ranging from paucimannosidic to complex tetra-antennary N-glycans.

## Methods

### Sample collection and grouping of cases

Tumor tissue samples were selected from consecutive cases to represent the main subtypes as defined by IHC, morphology, and tumor grade, with a minimum of 10 samples per group. Patient numbers and immunohistochemical profiles of each subtype are described in Table 1.

**Table 1:** Overview of breast cancer subtypes.

Breast cancer subtypes		Abbreviation	Grade	# Tissue samples	# Processed samples	Immunochistochemical markers			
						ER	PR	HER2	Ki67
Luminal (L)	Lobular	LA	1–2	12	12	** + **	** + **	**−**	Low
		LB	3	12	12		any		Moderate–high
	Mucinous	MUC		11	11	** + **	any	**−**	
	No-special-type	NST/LA	1	20	20	** + **	** + **	**−**	Low
		NST/LX	2	21	20		any		Moderate
		NST/LB	3	20	20				High
HER2 positive	ER-negative	HER2 +		12	12	**−**	**−**	** + **	Moderate–high
	ER-positive	LBH		12	12	** + **	any		Moderate–high
Triple negative breast cancer	AR-negative	TNBC		20	17	**−**	**−**	**−**	High
	AR-positive	LAR		10	9				High
Controls		CTRL		10	10				
			Total #	160	155				

Number of cases and controls with the IHC profiles are introduced for the individual subtypes. AR is tested mainly in TNBC; AR status was either positive or unknown for luminal and HER2 tumors.

The dataset represents tumors collected from 150 patients with breast cancer (median age 57 years) of various stages, tumor morphology, and size. The resection specimens were received from the operation theatre in a native state and the macroscopic evaluation was done immediately. Detailed clinical and histological data with the representative tissue histology images are listed in Additional file S1. Each group was determined by morphology and IHC profiles of diagnostic samples. Tumors were divided into three main groups, based on the three major IHC-determined phenotypes: luminal (ER-positive, HER2-negative), HER2-positive, and TNBC. The luminal HER2-negative group was further subdivided according to morphology into lobular, NST, and mucinous cancers. The first two mentioned types, which are the most common, were further divided based on grade and Ki-67 status. Lobular carcinomas grade 1 or 2 with low Ki67 index (≤ 20%) were labeled as lobular/luminal A (LA), while lobular carcinomas of grade 3 with Ki67 index above 20% were labeled as lobular/luminal B (LB). NST carcinomas were subdivided into luminal A (NST/LA)—grade 1, PR positive, Ki67 low, luminal B (NST/LB)—grade 3, Ki67 high (≥ 40%), and luminal indeterminate (NST/LX)—grade 2, Ki67 21–39%. Tumors not compatible with the above criteria (e.g. grade 2 tumors with high Ki67) were not included in the study). Mucinous carcinomas (MUC) were included without taking grade and proliferation rate into consideration. HER2-positive tumors were grouped into ER-positive (LBH) and ER-negative (HER). TNBC comprised ER-, PR- and HER2- negative tumors, and subdivided into luminal AR-positive (LAR) subtype and AR-negative (TNBC) subtypes.

All samples were retrieved from the Department of Pathology at Masaryk Memorial Cancer Institute (MMCI), Brno, Czech Republic^27^. The study was approved by the Institutional Ethic Committee of MMCI, reference number 2015/807/MOU (JID: MOU 61 772), 17 March 2015. The use of patient samples was ethically approved and a written informed consent was provided by all patients. The use of tissues complies with the Declaration of Helsinki and national legislation. The samples originate from 2013 to 2016. The tumor tissue samples were all from surgeries performed as the primary therapy with no prior treatments. Healthy tissue adjacent to tumor (control tissue) was also resected during surgery from 10 patients. All resected tissues were snap-frozen in liquid nitrogen and stored (− 196 °C) in the MMCI Biobank. Parallel tissue samples were fixed in formalin and routinely processed into paraffin wax blocks for diagnostic histopathology. All samples were randomized prior to sample preparation to reduce the impact of sample preparation and control batch effects, thereby providing an unbiased evaluation of the results.

### N-glycan release from tissue samples

If not stated otherwise, all chemicals were purchased from Merck (Rahway, NJ, USA) and purity was HPLC grade or higher. The protocol for N-glycan isolation was derived from Lattova et al.^28^. Frozen tissue samples (10–20 mg) were cut into small pieces with a sterile scalpel and washed five times with cold phosphate-buffered saline to remove blood residues. After removing the last liquid fraction, 300 μL chloroform, 150 μL methanol, and 37.5 μL deionized water were added to the tissue, which was vortexed and sonicated for 5 min. The sample was centrifuged at 615 rcf for 3 min, the supernatant was discarded and the tissue pieces were dried in a vacuum centrifuge for approximately 10 min. 100 μL water was added, incubated for 5 min at 95 °C, and cooled to room temperature. N-glycans were cleaved by adding 5 μL 100 mM ammonium bicarbonate and 0.3 μL PNGase F (NEB, Ipswich, MA, USA, 500,000 U/mL) and incubating for 3 h at 37 °C. The mixture was centrifuged at 750 rcf for 2 min, and the supernatant was collected in a new tube. Another 30 μL water was added to the tissue pieces, which were re-centrifuged (620 rcf, 2 min), and the supernatant was collected and pooled with the previous supernatant. The volume of the combined supernatants was reduced to ~ 50 μL by vacuum centrifugation at room temperature. The released N-glycans were then purified on Strata X-C 33 μm columns (Phenomenex, Torrance, CA, USA) as described previously^23^. Cartridges were washed with 3 × 800 μL methanol and 3 × 800 μL water. Samples were loaded on the column and eluted with 4 × 50 μL water. The released glycans were further purified using graphite SPE, reduced, and permethylated as described below.

### Graphite SPE

Graphite centrifugal columns (Harvard Apparatus, Hollister, MA, USA) were pre-equilibrate by washing three times with 250 μL aqueous solution with 85% acetonitrile (ACN) and 0.1% trifluoroacetic acid (TFA), then three times with 250 μL aqueous solution with 5% ACN (loading solution). To obtain glycans for samples, loading solution (250 μL) was added to each sample, the samples were vortexed, centrifuged for 1 min at 620 rcf and the supernatants were loaded onto the graphite columns. The samples were reloaded twice before washing the graphite sorbent twice with 250 μL loading solution. N-glycans were eluted with 200 μL solution with 30% ACN and 0.1% TFA (elution solution). The graphite columns were centrifuged for 10–15 s to equilibrate, left for 10 min for elution, and then centrifuged for 90 s. A second elution was performed with 200 μL elution solution in a single step. Centrifugal speed of 320 rcf for 2 min was used in all steps if not stated otherwise. The combined eluates containing N-glycans were dried in a vacuum centrifuge.

### Reduction and solid-phase permethylation

The purified N-glycans were reduced to their alditol forms by adding 10 μL aqueous 10 mg/mL solution of ammonium-borane complex and incubating at 60 °C for 1 h. After cooling to room temperature, excess ammonia-borane complex was disrupted by adding three 1 μL aliquots of glacial acetic acid at 1- h long intervals. Samples were then dried under vacuum and the borate salts were removed as their methyl ester by the addition of three 100-μL aliquots of methanol for 1 h, followed by drying in a vacuum centrifuge. The N-linked glycans were then permethylated in N,N-dimethyl formamide (DMF) as previously described^29^. Briefly, microscale spin-column reactors were prepared by first loading the empty columns with sodium hydroxide beads re-suspended in acetonitrile. The reactors were then washed with DMF. The dried N-linked glycan samples were re-suspended in 65 μL DMF/35 μL methyl iodide/5 μL water, loaded onto a microscale column, and incubated for 20 min at room temperature. Next, samples were centrifuged and the second aliquot of methyl iodide (25-μL) was added. Subsequently, the reaction solutions were reapplied to the reactors for a second 20-min period before centrifugation. The permethylated analytes were recovered from eluates by a liquid/liquid extraction procedure using chloroform and repeatedly washed with 0.5 M sodium chloride and water. Following extraction, the chloroform layer containing the permethylated N-linked glycans was dried in a vacuum centrifuge and stored at − 20 °C until measurement.

### MALDI-MS measurements

First, the samples were reconstituted in 5 μL 50% methanol. One μL of each sample was spotted on a polished steel MALDI target and allowed to dry. Subsequently, 0.5-μL 2,5-dihydroxybenzoic acid matrix solution (10 mg/mL in 50% methanol, supplemented with 1 mM sodium acetate) was added to the samples and air-dried. Each sample was spotted in triplicate. Mass-spectrometric analyses were performed on the MALDI-TOF/TOF instrument UltrafleXtreme™ (Bruker Daltonics, Germany) in the reflectron positive-ion mode. Prior to all analyses, the instrument was calibrated with the peptide calibration standard mixture (Bruker Daltonics, Germany). Mass spectra were acquired using the FlexControl software; 5000–10000 individual mass spectra were averaged for each spot. Each spectrum was internally calibrated with a minimum set of four selected monoisotopic peaks corresponding to known glycan compositions calculated as [M+Na]^+^ for reduced permethylated derivatives (H2N2F1 1157.6038; H5N2 1595.8139. H5N4 2068.0665; H5N4F1 2260.1558; S1H5N4F1 2621.3294; S2H5N4 2808.4139; S1H6N5F1 3070.543; S3H6N5 3618.81361; S3H6N5F1 3792.9029). The structural assignment of N-glycans was derived from their molecular masses and from LIFT tandem mass spectrometry (MS/MS) experiments. The branching, fucose position, and presence of bisecting moiety were performed through additional MS/MS experiments using phenylhydrazine-derivatiation of desialylated glycan pools^30,31^. MALDI mass spectra were processed in FlexAnalysis software (Bruker) using a method created directly for permethylated glycans. First, the mass-spectral baseline was subtracted (TopHat) and spectra were smoothed (using the Savitzky-Golay algorithm with width 0.08 m/z; 5 cycles). The peak lists were found using the following parameters: SNAP was used as the peak detection algorithm, with average composition corresponding to average permethylated N-glycan (C_1_O_0.5_H_1.6_N_0.05_)_n_Na; quality factor threshold was set to 50.

A Python script written for this purpose (see Additional file S2) was used to extract the areas of isotope envelopes with monoisotopic *m/z* values corresponding to masses in a reference N-glycan library. The N-glycan library (see Additional file S3) contains 98 generally recognized N-glycan masses and their tentative structures previously reported^17,32^ or characterized in this study. The first peak was searched within a mass window around reference masses ± 0.2 Da, while the mass error (ref. mass—measured mass/ref. mass) of the assigned peak was calculated and taken into account in the ongoing database and peak list iteration: the center of the mass window was shifted by the mass error to compensate for mass shift in the range of higher m/z values. The area under the curve (AUC) of each N-glycan peak was normalized by the sum of all N-glycan peak areas (100%), yielding a normalized peak area (AUCn). Only N-glycan signals with S/N > 3 were included in the final dataset and exported as a .csv file (see Additional file S4).

### Data processing and statistical evaluation

The exported datasets were submitted to statistical analysis based on the differences in glycan signal with respect to the listed clinical data (tumor classification, size, Ki67 etc.). The reproducibility-optimized test statistic (ROTS) software package in R was performed^33^. In parallel, the Wilcoxon rank sum test with continuity correction was performed.

Further data analysis was conducted in MS Excel with the following settings: signals with p-value lower than 0.01 for both tests, false discovery rate (FDR) < 0.01 and with at least 50% occurrence in the group with elevated N-glycan signal were considered as significant. Subsequently, Venn diagrams were created, where the size of a tentative N-glycan structure corresponds to the mean ROTS score (absolute values) for each N-glycan signal in comparisons in which the N-glycan signal was found to be significant: Three different structure sizes were used; average ROTS score higher than 8 (the largest structures), between 4 and 8, and lower than 4 (the smallest structures).

### Ethics approval and consent to participate

The study were approved by the Ethics Committee of Masaryk Memorial Cancer Institute (No. 2015/807/MOU, JIT MOU61772). All patients provided written informed consent.

## Results

### General considerations

Although numerous studies have reported that unusual protein glycosylation is coincident with different types of malignancy, the altered glycosylation pathways have not yet been adequately documented and understood. The overall glycosylation profile of a cell, normal (control) or malignant, is a product of the complex molecular machinery with dozens of enzymes further affected by the cell’s local environment and the subsequent changes in substrate and enzyme availability^34^. Since breast cancer is a heterogeneous disease with a variety of histological and clinical characteristics, some diversity in N-glycan profiles across the individual subtypes can be expected^35^. The goal of the study presented here is to better characterize glycosylation patterns associated with different subtypes of breast cancer using clinically distinguishable and documented cases. Glycosylation-oriented searches for breast cancer markers have been actively pursued for well over a decade, yet it remains a challenging task because of the possible variations due to patient’s age, ethnicity and different pathophysiological conditions^36,37^. Besides these inherent complications, there are methodological discrepancies within different types of measurements, which could explain some inconsistent results from different laboratories. Our profiling method of choice has been MALDI-MS measurement of permethylated N-glycans which makes it feasible to compare quantitatively a large number of samples for further statistical analysis.

Permethylation of oligosaccharides is an established derivatization technique^29^, noted for its inclusion of a wide range of glycans and stabilization of sialic acid residues and fucosyl substituents that could otherwise be labile during MS^25,38^. This is particularly important for detecting multi-antennary structures. While sample preparation through permethylation is procedurally more complex than some other approaches, the permethylation and MALDI-MS methods can be multiplexed and potentially automated^26^. However, it must be recognized that this approach is not optimal for distinguishing all structural isomers, such as α-2,3- from α-2,6-linkages of sialic acids. These structural entities that may be functionally important^39,40^ are being addressed by the development of alternative methodologies^41–43^ and have also been applied to patient samples^44,45^.

Whereas most previous glycomic studies of breast cancer were carried out using serum or plasma, much less information is available on tissue specimens, which are more methodologically demanding samples. In this study, we used our local breast cancer cohort of 150 cases from which 145 tumor samples were processed together with 10 control tissue specimens adjacent to tumors. Four specimens could not be fully evaluated due to methodological difficulties in tissue extraction. The different cancer subtypes were determined based on state-of-the-art IHC and histological methods. In total, 92 N-glycan signals could be detected, from which 59 were present in more than 50% of samples. Statistical evaluation of signal proportions was performed on five subgroups: (1) luminal-mucinous (MUC); (2) luminal-lobular (L); (3) luminal-no-special type (NST); (4) HER2 positive (HER2 +), and (5) triple-negative breast cancer (TNBC). The sixth group represents control tissue, i.e. the seemingly healthy tissue samples adjacent to the tumors. The N-glycosylation features, were considered together with their respective signal intensity.

### Glycosylation features and major N-glycan groups across breast cancer subtypes

**Figure 1:**
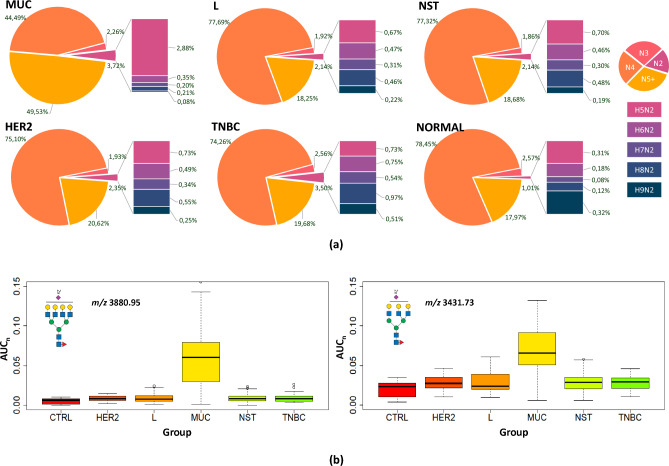
Distribution of major N-glycan groups in all tissue types. (**a**) Distribution of N-glycans containing following units of N-acetylglucosamine: (i) N2—two units (corresponding mostly to high-mannose N-glycans), (ii) N3—three units (~ hybrid), (iii) N4—four units (~ biantennary), and (iv) N5 +—five and more units (~ 3- and 4-antennary) in breast cancer subtypes and control tissue samples. (**b**) Box plots of normalized peak area (AUC_n_) of representative N-glycans (*m/z* 3880.95 and 3431.73) in the subgroups.

Changes in the extent of N-glycan branching, oligomannose occurrence, and the extent of sialylation/fucosylation have been recognized as generally associated with breast cancer progression^46,47^. In our study, we identified the overall distribution of major N-glycan groups in all tissue types, summarized in Fig. 1A. The major groups of glycans were interpreted with respect to the number of GlcNAc units, with high mannose N-glycans containing two units, hybrid N-glycans three, bi-antennary structures four, and complex N-glycans five GlcNAc units. At first glance, the MUC subtype is different from all others (Fig. 1B). A 2.5-fold increase in tri- and tetra-antennary glycans is readily observable in comparison to the other subtypes.

Another trend visible in Fig. 1A is an increase in oligomannose N-glycan structures (N2—two units of N-acetylglucosamine). These N-glycans, especially their under-processed structures carrying seven to nine mannose units, are not well documented but have been associated with cancer development^31,48,49^. In our study, their occurrence increases from control tissues (1%) to more aggressive breast cancer subtypes. In TNBC, the occurrence of oligomannose N-glycans increased 3.5-fold. An even higher abundance (3.7%) was observed in the MUC subtype, while the distribution of individual N-glycan structures was shifted towards structures with a lower number of mannose residues.

Changes in sialylated and fucosylated N-glycan structures (see Additional file S5) were in the order of percentage units. Except for the MUC-associated N-glycans (80% of sialylated N-glycan species), the sialylation level was between 73.5% (control tissue) and 76.9% (L). The variation of total fucosylation was slightly higher: N-glycans in MUC carried the highest fucosylation level (86%). The lowest mean fucosylation level was in TNBC (76.1%), followed by control (77.7%), HER2 (79.3%) and NST and L subtypes (81.1 and 82.6%, respectively).

### Changes in individual N-glycan structures

#### MUC subtype compared to other subtypes

**Figure 2:**
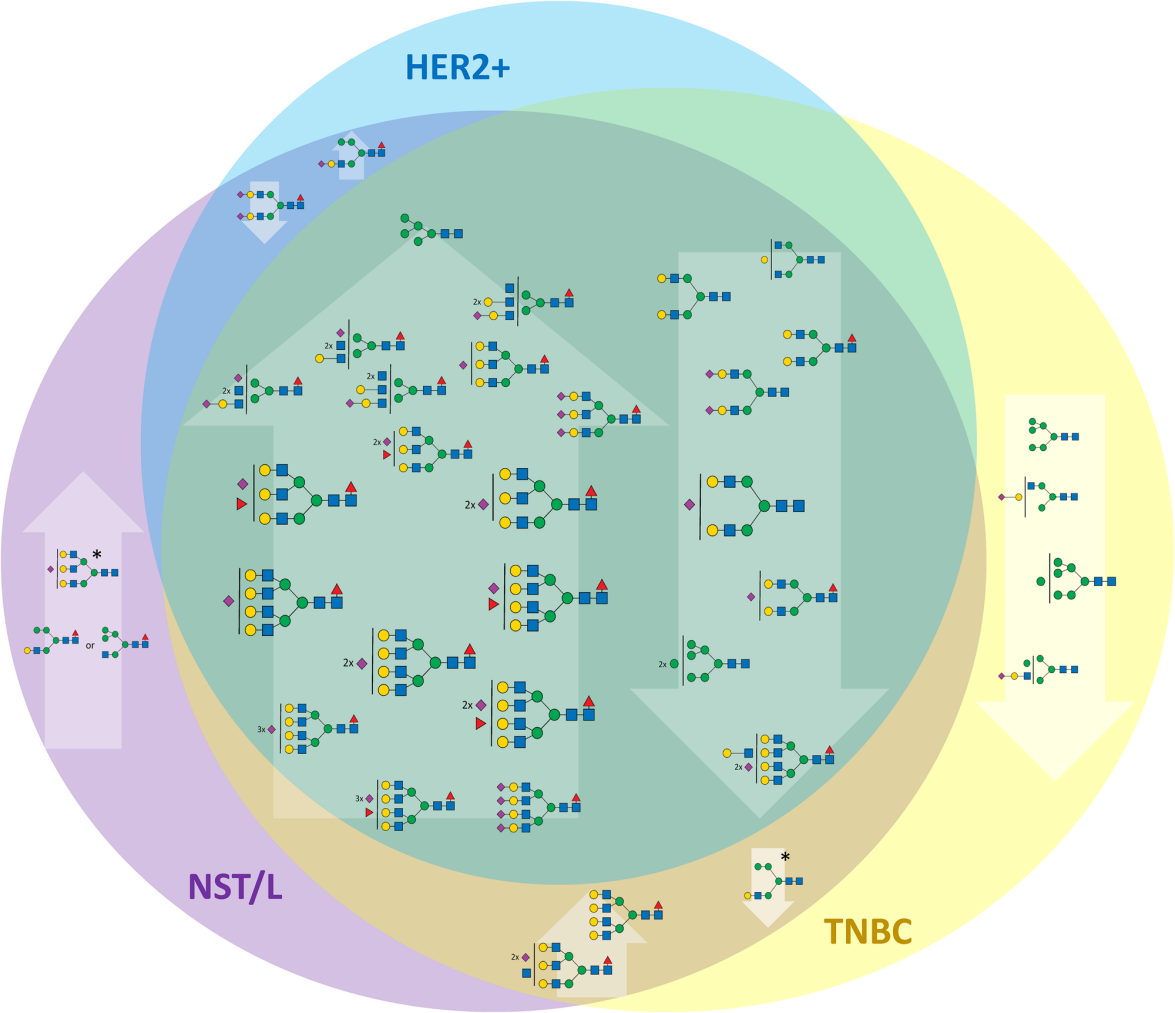
Changes of the N-glycan signals in MUC subtype. Venn diagram showing the tentative structures corresponding to N-glycan signals elevated (upward arrows) or decreased (downward arrows) in the MUC subtype compared to TNBC, HER2, and NST/L subtypes. The size of the tentative N-glycan structure corresponds to the mean reproducibility-optimized test statistic (ROTS) value for each N-glycan signal.

As described in the previous section, N-glycan profiles stood out in branching of N-glycans as well as overall fucosylation and sialylation levels (Fig. 2*, *Additional files S6 and S7). At the individual N-glycan levels, 16 signals were increased and 6 were decreased in the MUC subtype compared to all other groups (p-value < 0.01 and FDR < 0.01, occurrence at least 50% in the group with elevated N-glycan signal). As expected, the elevated glycans were mostly fucosylated (18/19), sialylated (16/19), multi-antennary with 3 or 4 antennae (14/19), and hybrid glycans (2/19). On the other hand, decreased glycans (9 structures) were bi-antennary glycans with various fucosylation and sialylation levels, except for two signals at *m/z* 4330.18 and 2208.11 (corresponding to H8N7F1S2 and H8N2); their lower occurrence correlated with an increase in their multiply branched counterparts. The complete list of statistically significant N-glycan signals for each group including p-values and summary formulas is attached in Additional file S7. As mentioned earlier, the distinct difference in N-glycan composition, including the high level of tri- and tetra-antennary N-glycans, presumably reflects the histological and molecular composition of the tumor. Due to the large number of differentiating N-glycans, the results concerning MUC are not included in further discussion, unless stated otherwise.

#### TNBC subtype compared with other subtypes

**Figure 3:**
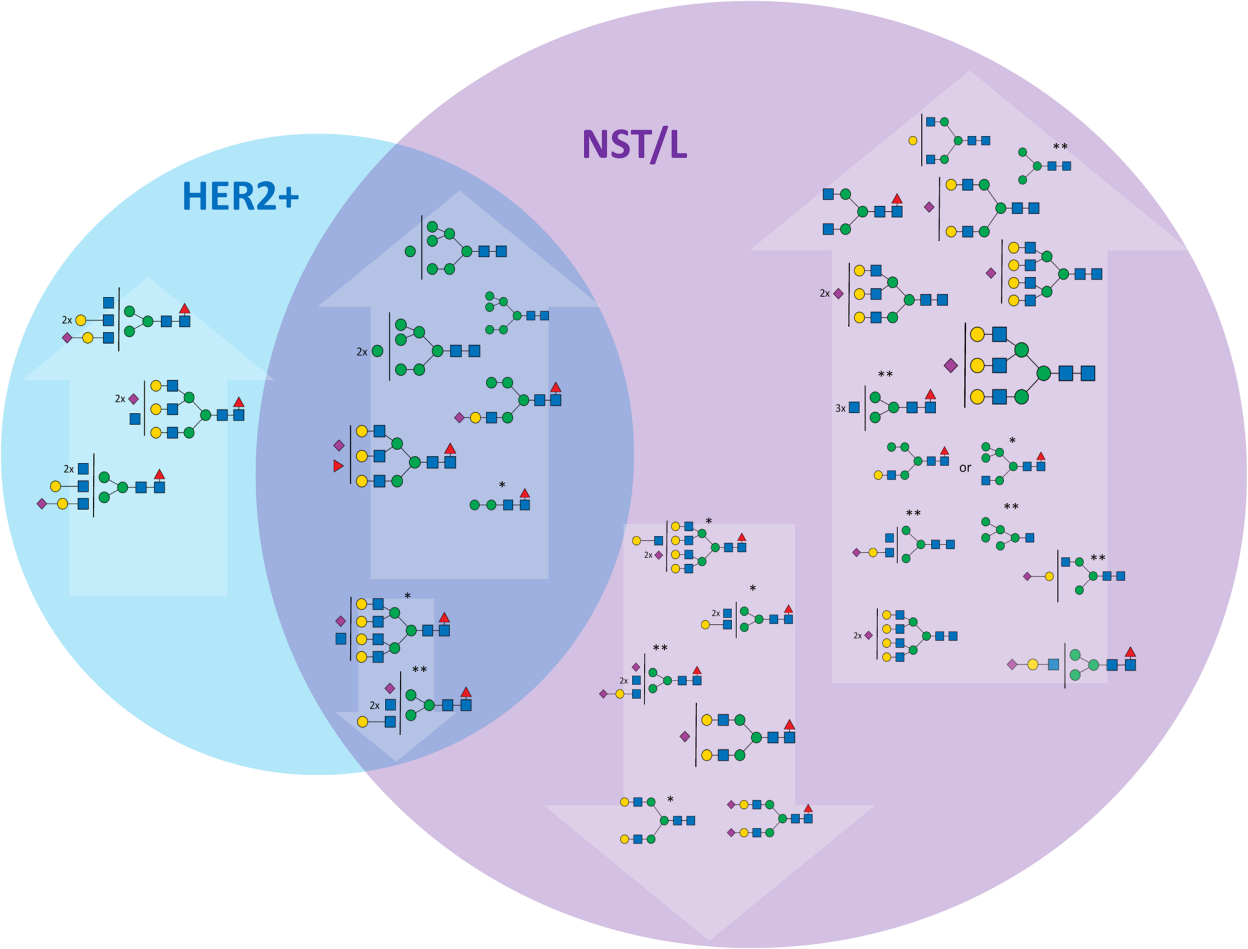
Changes of the N-glycan signals in TNBC subtype. Venn diagram showing tentative structures corresponding to the significantly elevated N-glycan signals (in arrows pointing up) and decreased (in arrows pointing down) in the TNBC subtype, in comparison to HER2 and NST/L. The tentatively assigned N-glycan structures correspond to the mean ROTS value for each N-glycan signal (* and ** marked N-glycans do not have statistically significant changes in comparison to NST vs TNBC or L vs TNBC, respectively).

As seen in Fig. 1A,B, the other subtypes shared a similar distribution of N-glycan groups differing at most in the high-mannose glycans. In TNBC, glycans containing two N-acetylglucosamine units, corresponding mostly to high-mannose glycans, represent 3.5% of the total N-glycan amount compared to L and NST (2.1% of total N-glycan) and HER2 (2.3%). Moreover, the distribution of individual high-mannose N-glycans is shifted towards those with a higher number of mannose residues. Statistical analysis of the individual N-glycan signals (Fig. 3) showed that N-glycan signals were higher in TNBC than other subtypes (including control and MUC). Three of these signals were seen at *m/z* 2208.11, 2004.01, and 1799.91, corresponding to oligomannose structures H8N2, H7N2, and H6N2 (Additional files S8 and S9). It suggests that the downregulation of a Golgi mannosidase results in an increase of high-mannose N-glycans in breast cancer^35,50^. These data are also in good agreement with the increase in high-mannose N-glycan structures (H8N2, H9N2) in TNBC observed by MALDI mass spectrometry imaging^48^. Additionally, three signals were found to be increased in comparison to other subtypes (HER2, NST, and L), with signals at *m/z* 2172.10, 2376.20, and 3244.64, corresponding to fucosylated and sialylated H4N3F1S1, H5N3F1S1 and H6N5F2S1 structures. The list and boxplots of the above-mentioned N-glycan signals in TNBC compared to all subtypes are represented in Additional files S8 and S9. Other N-glycan signals altered in comparison to HER2 + (N = 5), NST (N = 21), or L (N = 17) subtypes can be found in Additional file S8.

### HER2/NST/L subtypes compared with other subtypes

**Figure 4:**
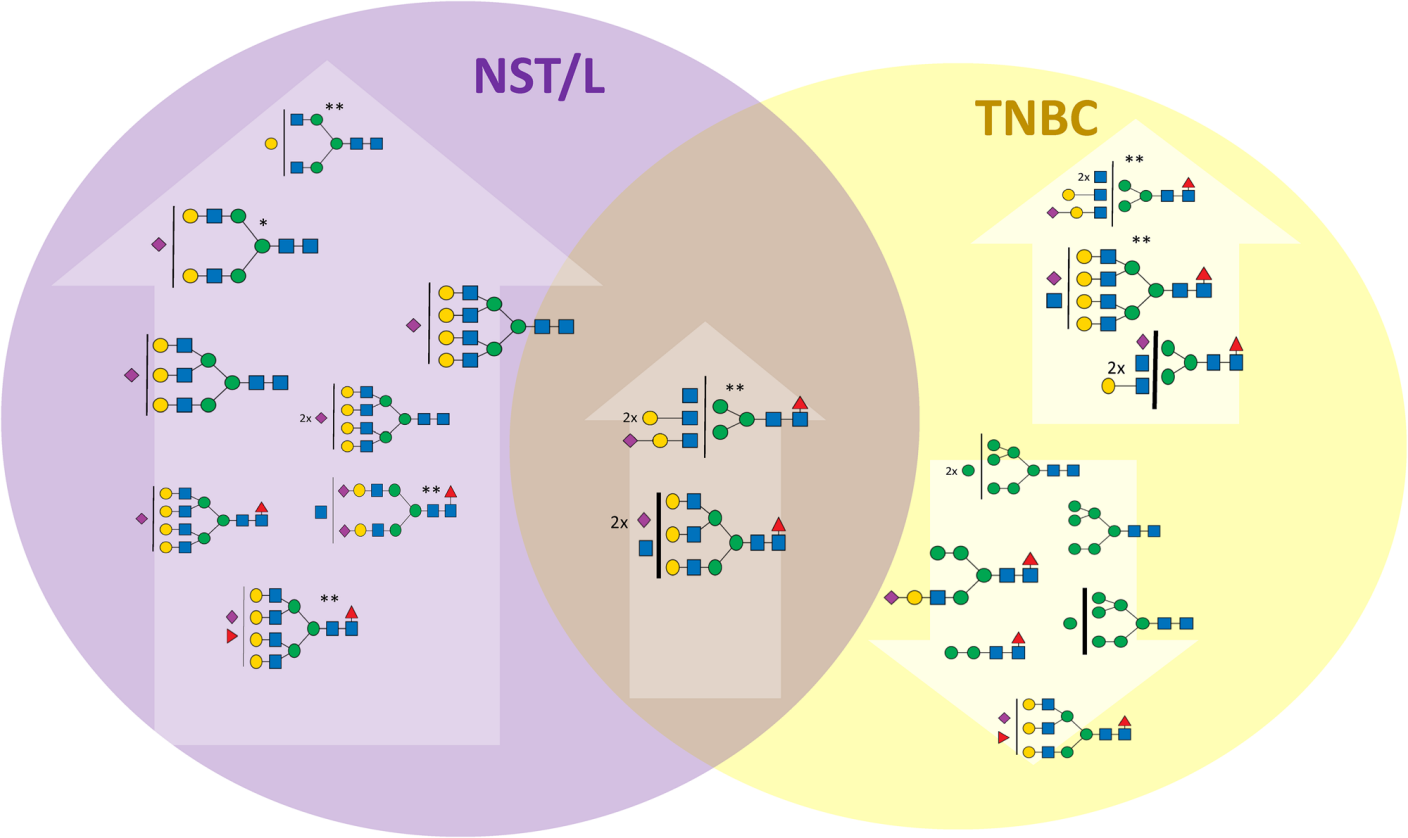
Changes of the N-glycan signals in HER2 subtype. Venn diagram showing tentative structures corresponding to the significantly elevated N-glycan signals (in arrows pointing up) and decreased (in arrows pointing down) in the HER2 subtype, in comparison to TNBC and NST/L. The tentatively assigned N-glycan structures correspond to the mean ROTS value for each N-glycan signal (* and ** marked N-glycans do not have statistically significant changes in comparison to NST vs HER2 or L vs HER2, respectively).

Comparison of the remaining three tumor type groups (HER2, NST and L) did not identify specific changes. Here, subtyping relies in all cases on structurally related sets of N-glycan signals: in HER2 tumors, only N-glycan signals at *m/z* 3315.68 and 3676.85 (mono- and disialylated H6N6F1S1 and H6N6F1S2) were elevated compared to both TNBC and NST or L tumors (Fig. 4, Additional files S10 and S11). Additionally, three singly sialylated N-glycan signals at *m/z* 2896.47, 3345.69, 2447.24 (H6N5S1, H7N6S1, H5N4S1) and two di-sialylated N-glycan signals at *m/z* 3257.64, 370,687 (H6N5S2, H7N6S2), all with no fucose substitution, were increased compared to the NST and L subtypes. The N-glycan signals at *m/z* 2172.10 and 2376.20 (H4N3F1S1 and H5N3S1F1) significantly decreased only in comparison with TNBC are shown in Fig. 4.

**Figure 5:**
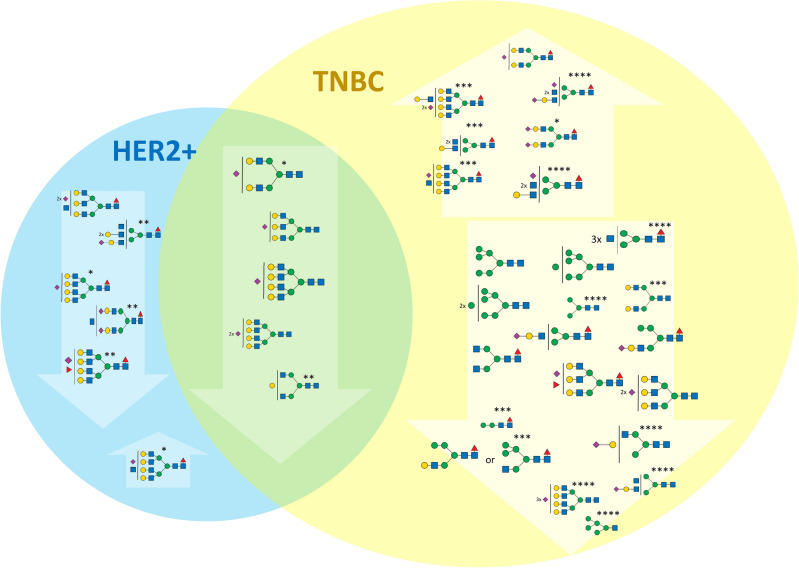
Changes of the N-glycan signals in NST subtype. Venn diagram showing tentative structures corresponding to the significantly elevated N-glycan signals (in arrows pointing up) and decreased (in arrows pointing down) in the NST subtype, in comparison to TNBC and HER2 + . The tentatively assigned N-glycan structures correspond to the mean ROTS value for each N-glycan signal (*, **, ***, and **** marked N-glycans do not have statistically significant changes in comparison to NST vs HER2, L vs HER2, NST vs TNBC, and L vs TNBC, respectively).

Finally, the group of N-glycans that were decreased in NST and L compared to TNBC and HER2 samples, apart from the above-mentioned signals corresponding to N-glycan signals at *m/z* 2447.24, 2896.47, and 3257.64 (H5N4S1, H6N5S1, and H6N5S2) also included N-glycan signals *m/z* 3345.69 and 3706.87 that correspond to tetra-antennary H7N6S1 and H7N6S2 (Fig. 5, Additional files S12 and S13). The unique shared feature of these structures is the absence of a fucose unit and the presence of sialylation. N-glycan profiles of L and NST subtypes show high similarity. The statistically significant N-glycans for differentiation of these subtypes were the complex mono-fucosylated signals at *m/z* 2621.33 and 4330.18 (H5N4F1S1 and H8N7F1S2) that were increased in L compared to NST and *m/z* 2662.36 and 3023.53 (H4N5F1S1 and H4N5F1S2) that were decreased in L compared to NST (Additional file S12).

### Control breast tissue N-glycan profiles

**Figure 6:**
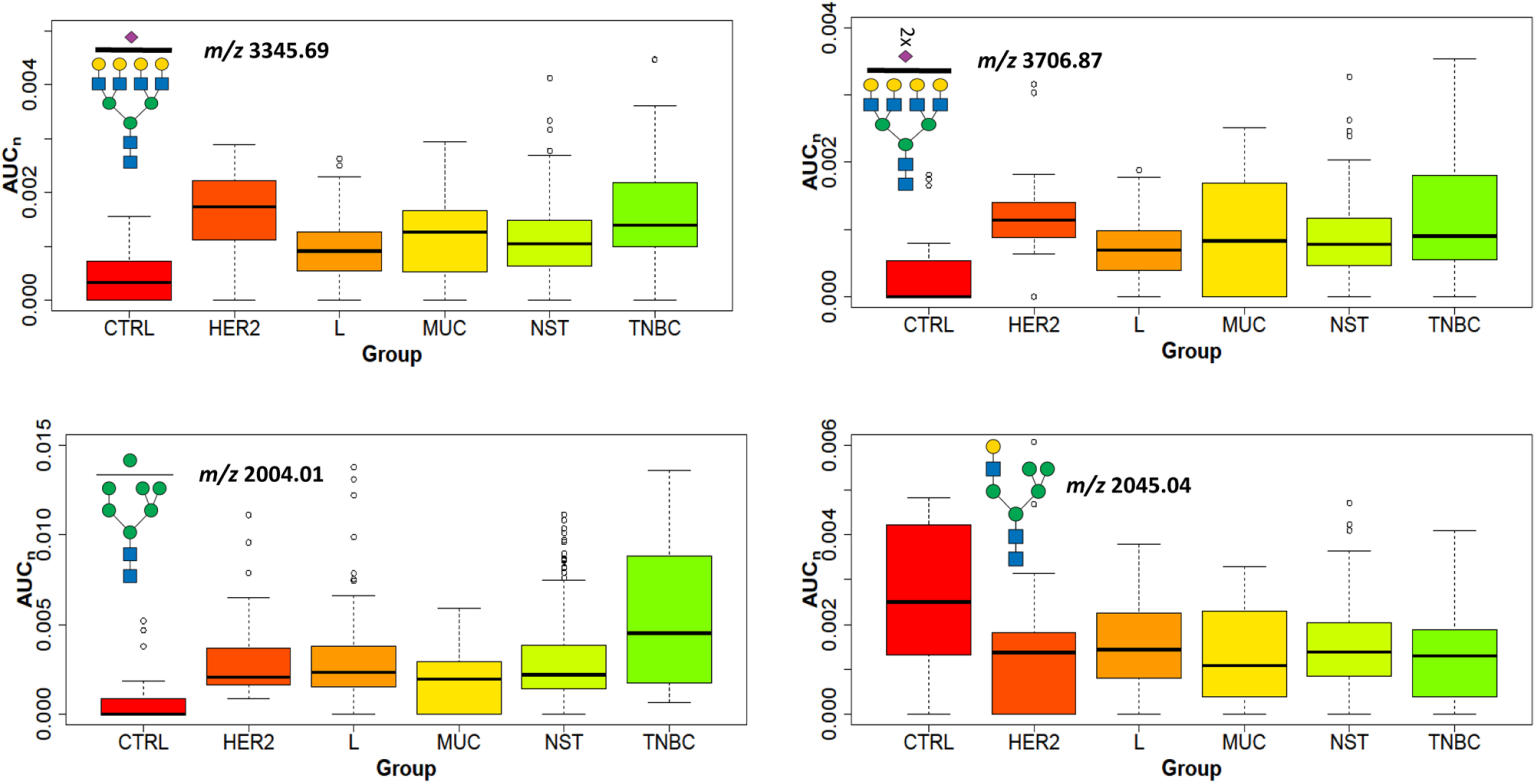
Changes of the N-glycan signals in controls. Boxplots with the normalized peak area (AUC_n_) of representative N-glycans decreased (*m/z* 2045.04) or increased (*m/*z 3345.69, 3706.87, 2004.01) in cancer tissues compared to control tissues.

N-glycans from healthy tissues adjacent to the tumor (controls) were compared with breast cancer subtypes (L, MUC, NST, HER2 +, and TNBC). Groups of N-glycans were identified that could distinguish controls from tumor tissues, moreover, some glycans were unique for one or more subtypes in these comparisons (Fig. 6*, *Additional files S7, S8, S10, S12).

The increased signal levels of tetra-antennary N-glycans decorated with fucosylation or sialylation (*m/z* 3345.69, 3693.87, 3706.87, 3880.95, and 4242.13) or high mannose glycan (*m/*z 2004.01) were found in all cancer subtypes analyzed in this work. In contrast, the bisecting (*m/z* 2505.28) and bi-antennary complex N-glycans (*m/*z 2045.04, 2117.09, and 2692.37) had decreased signals. The presence of a bisecting GlcNAc unit in glycans was observed to affect growth factor signaling and tumor progression in cell cultures^51,52^, and inhibits epithelial-mesenchymal transition (EMT) in hypoxic tumor cells^52^. The decrease of bisecting GlcNAc and the corresponding downregulation of N-acetylglucosaminyltransferase (MGAT3) was shown in an independent study using cell lines^53^.

Uniquely, only the MUC subtype, which remains outside the other described subtypes, had significantly decreased signals of N-glycans at *m/z* 1881.97, 2086.07, 2202.11, 2260.15, 2621.33, and 2808.41 and an increased N-glycans at *m/z* 4055.04 compared to control tissues. In contrast, N- glycans at *m/z* 1799.91 and 2208.11 (both are high-mannose glycans) were increased and glycans at *m/*z 2301.18 and 2866.46 (bisecting glycan) decreased in all cancer subtypes except for the MUC subtype compared with controls. The increased signal of N-glycans at *m/*z 983.51 was observed solely in TNBC and *m/z* 4691.35 in HER2 +, compared to control tissues. The NST subgroup is distinguished by the lack of two N-glycans at *m/z* 1187.61 and 1432.74 that were increased in the other subgroups (further differences in N-glycan signals are provided in Additional files S7, S8, S10, S12).

### Connection of N-glycans with clinical parameters across breast cancer subtypes

Besides grouping tumors into the major subtypes, histological aspects allow an orthogonal data analysis, as several subtypes include samples with heterogeneous parameters. For instance, luminal subtypes (LA, LB, MUC, and NST) contain samples with very different signals of proliferation. The effect of tumor size, proliferation marker Ki67, lymph node metastases, and lymphovascular invasion on N-glycosylation profiles were also investigated.

### Tumor size

N-glycan cancer tissue profile data from all subtypes were divided into two groups based on tumor size (Additional file S1 with clinical data). The group with smaller tumors (pT1; ≤ 2 cm) contained 67 samples, and the group with larger tumors (pT2 and pT3, > 2 cm) contained 77 samples. At the signal of individual N-glycans, 8 signals were significantly altered; 7 were increased in pT2 + pT3 (*m/z* 1595.81, 1606.83, 1799.91, 1851.96, 2004.01, 2056.06, and 4603.30) and one was decreased (*m/z* 2621.33; Additional file S14). Except for a single tetra-antennary N-glycan (*m/z* 4603.30 corresponding to H7N6F1S4), all other elevated signals were low molecular size, represented by (i) paucimannose, (ii) high-mannose (oligomannose)**,** or (iii) galactosyl bi-antennary N-glycans.

**Figure 7:**
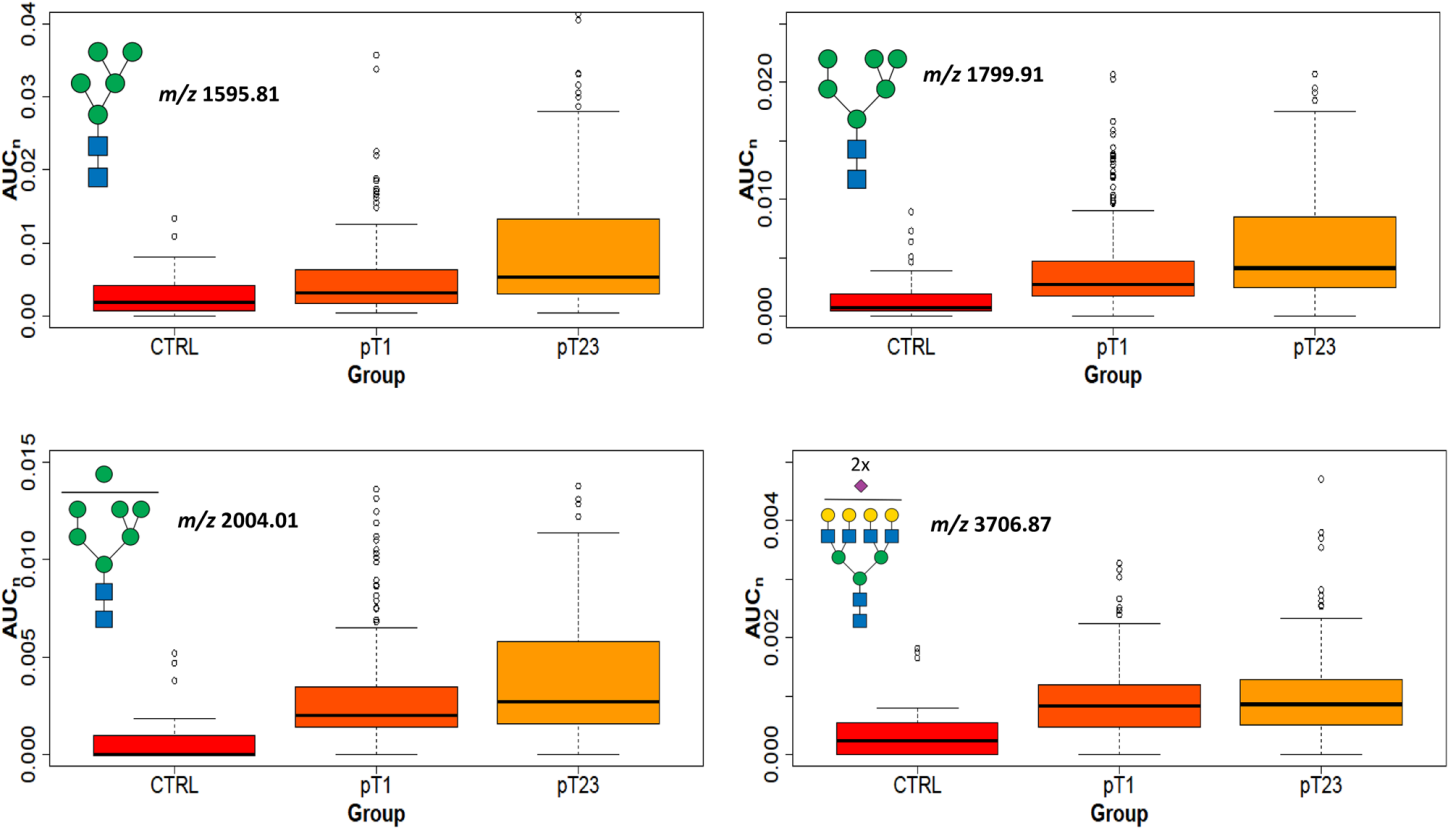
Tumor size. Boxplots of normalized peak area (AUC_n_) of the high-mannose and complex N-glycans (*m/z* 1595.81, 1799.91, 2004.01, and 3706.87) in tumors (pT1, and pT2 + pT3) compared to control samples (CTRL).

The comparison of control samples with either tumor samples pT1 or pT2 + pT3 provided 22 significantly increased and 11 decreased N-glycans (Additional file S14). All increased glycans were divided into two groups: (i) less or partially processed (high mannose and hybrid types with lower *m/z*), and (ii) fully processed (the complex type with three- or tetra-antennary structures detected at higher *m/z*). The quantity trends of four significant N-glycan signals chosen from both groups (high mannose and tetra-antennary) present in control and tumor samples are shown in (Fig. 7). These results demonstrate that N-glycan structures can distinguish control and tumor tissues. The presence of paucimannose and oligomannose N-glycans in tumor tissues suggests an incomplete glycosylation process with failure to undergo further glycosylation in the Golgi. This suggests that these less-processed N-glycans were involved earlier in the biological processes without extensive additional modifications in larger tumors (Additional file S14). Moreover, Driouich et al*.* reported that high-mannose glycans are important for protein folding, protecting the proteins against degradation during intracellular transport^54^, and can support cell growth.

### Cancer grade

**Figure 8:**
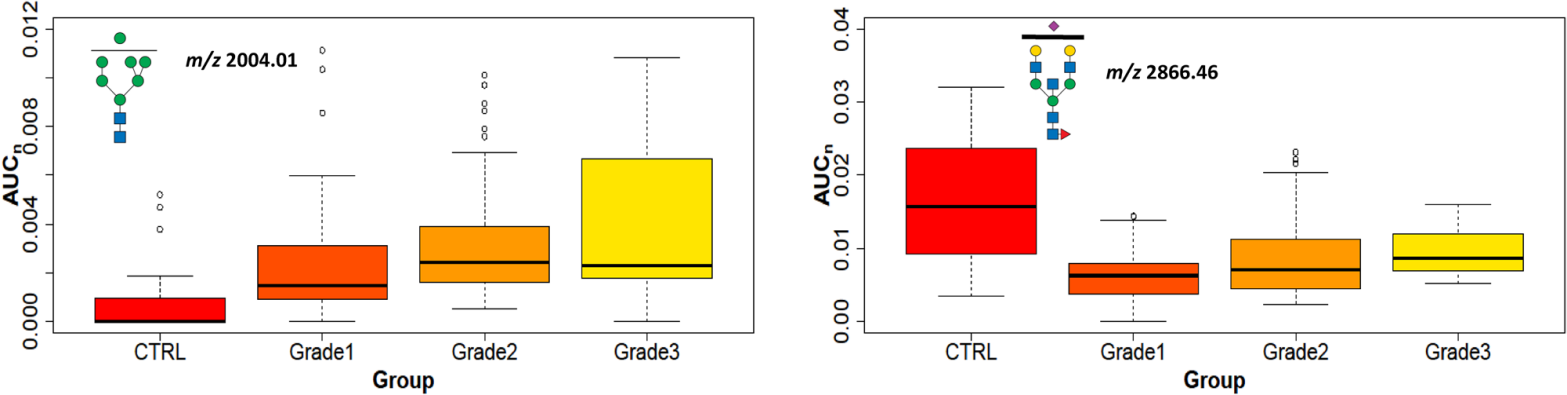
Cancer grade. Boxplots of normalized peak area (AUC_n_) of the representative statistically significant N-glycans in NST cancer tissue with *Grades 1–3* and control tissues.

Breast cancers are also classified according to pathological grade, reflecting cytological abnormalities and proliferative appearance: *Grade 1* shows similarity to normal breast cells, usually growing slowly; *Grade 2* shows higher cytological abnormality and grows faster; and *Grade 3* shows extreme cellular abnormalities and usually grows even more rapidly. Our cohort contained 20 NST cancer samples from each grade and 10 control tissue samples. *Grade 1* tumors showed an increase in 7 and a decrease in 11 N-glycans; *Grade 2* showed increases in 18 and decreases in 10; and *Grade 3* contained increased signals of 15 and a decrease of 8 N-glycan signals compared to control tissues (Additional file S15). Each grade showed 5 common increased N-glycan signals (*m/z* 1799.91, 2004.01, 2208.11, 3023.53, 3693.87) and 7 decreased (*m/*z 2097.08, 2127.09, 2301.18, 2505.28, 2692.36, 2866.45, 3315.68) compared to control samples. Principally, three high-mannose glycans are increased in *Grades 1* and *2* (*m/z* 1799.91, 2001.01, and 2208.11), and three other high-mannose glycans are increased in *Grade 3* (*m/z* 1157.60, 1350.69, and 1432.74). These increased glycans predominantly contained complex three- or tetra-antennary structures with higher numbers for *Grades 2* and *3* (Additional file S15). Tetra-antennary N-glycan *m/z* 3345.69 had increased signals in *Grade 2* and *3* tissues compared to controls, whereas the bisecting N-glycan *m/z* 2866.45 was decreased in all grades. This distinction could be due to the different cellular compositions of non-tumor tissues, as they contain little epithelium with more collagen and adipocytes. These N-glycan trends are shown in Fig. 8.

### Proliferation

**Figure 9:**
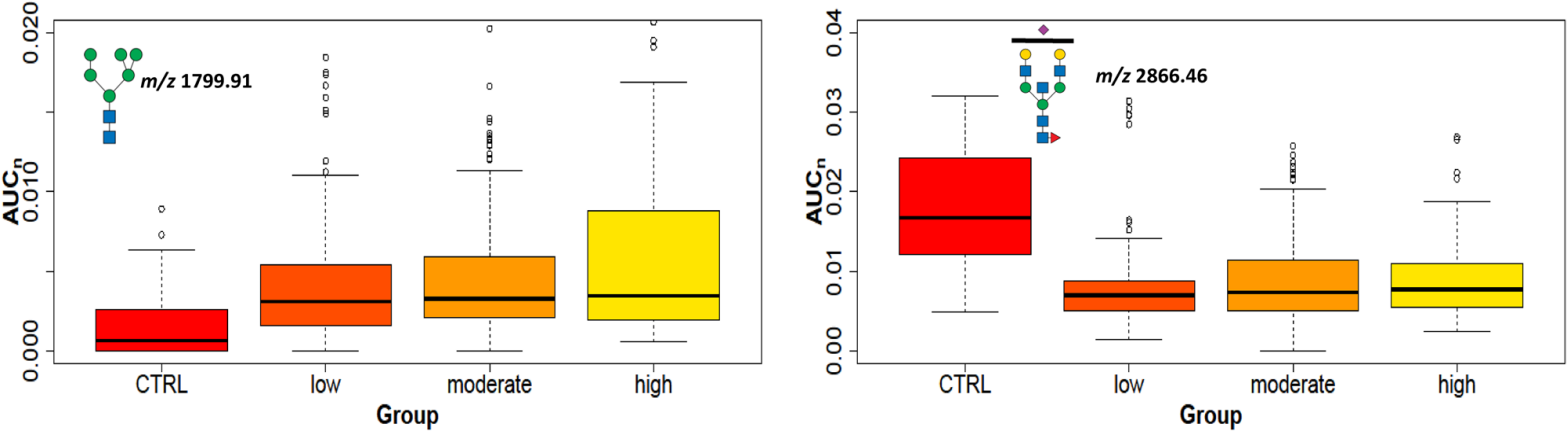
The proliferation marker Ki67. Boxplots of normalized peak area (AUC_n_) of representative statistically significant N-glycans in cancer tissues with Ki67 low, moderate, and high levels compared to control tissues.

**Figure 10:**
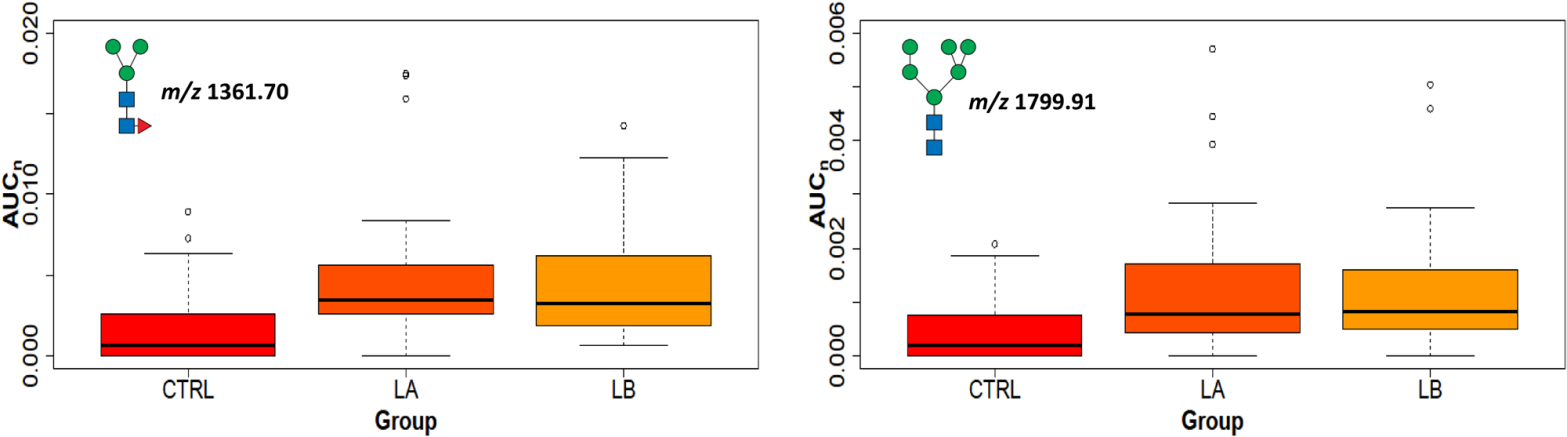
The intra-subgroup comparison of LA and LB. Boxplots of normalized peak area (AUC_n_) of the statistically significant high-mannose N-glycans LA and LB tumor tissues compared to control tissues.

Ki67 is a nuclear protein associated with cell proliferation and is an established prognostic and predictive cancer indicator. Tumor samples were classified into three groups: low (Ki67 < 20%), moderate (20–60%), and high Ki67 level (> 60%). High-mannose glycans were increased in NST compared to control tissues. The tumors with low and medium levels of Ki67 had 3 N-glycans with increasing trends (*m/z* 1799.91, 2004.01, and 2208.11), and 3 additional glycans were in tumors with a high level of Ki67 (*m/z* 1157.60, 1187.61, and 1432.74). These glycans with high mannose units detected in the most proliferative tissues were in good agreement with the literature^22^. Another type of N-glycans increased in NST tissues with Ki67 levels are complex tetra-antennary N-glycans that are mostly fucosylated on the core; but are also present on the antennae together with sialylation (occurrence from 1 to 4 fucosyl and sialyl units, for example, *m/z* 3519.78, 3706.87, 4068.04, 4603.30). On the other hand, the hybrid types of glycans (*m/z* 2045.04, 2097.08, 2243.14, 2301.18) and bisectin types (*m/z* 2505.28, 2866.46) were decreased in comparison with the controls (Fig. 9 and Additional file S16). In accordance with Grades 1–3 and Ki67, tetra-antennary or high mannose N-glycans (e.g. *m/z* 2004.01) were increased but bisectine glycans (e.g. *m/z* 2866.46) were decreased (Fig. 8). This means that these types of glycans can distinguish tumor tissues with elevated grade and Ki67 from non-tumor tissues. The comparison of lobular tumors (LA and LB) with controls shows three statistically increased bi- and triantennary N-glycans (*m/*z 2982.50, 3070.55, 3431.73) with fucosylation at the core at any level of Ki67 (Additional file S16). These results are in accordance with data in which fucosyltransferases, responsible for core fucosylation, were found to be elevated in aggressive breast carcinoma cells^55,56^. In contrast, the intra-subgroup comparison between LA and LB (cells in LB divide faster than in LA) N-glycans did not show any statistically significant signal changes (e.g. high mannose glycans *m/z* 1361.70, 1799.91, Fig. 10).

### Lymph node metastases

Cancer metastasis is commonly caused by invasion of primary cancer cells through the lymphatic nodes. Cancer progression and poor survival are indicated for breast cancer patients with positive sentinel lymph nodes^57^. We classified tumors into three groups: pN0 (without lymph node metastases), pN1 (with one to three lymph node metastases), pN2, and pN3 (with four to nine or more lymph node metastases, collectively designated pN_2-3_), Additional file S1. The statistically significantly increased N-glycan signals in tissues of patients with lymph node metastases (pN2-3) compared to pN0 in the NST subgroup were high mannose (*m/z* 1595.81, 1799.91, 2004.01, 2208.11) and hybrid type glycans (*m/z* **1606.83**, 1998.01, 2202.11, **2662.36**, **3023.53**; bold *m/z* numbers mark core fucosylated glycans). The same comparison was done for all, breast cancer subgroups, except mucinous, when signals for hybrid (*m/z* **2172.10**) and tetra-antennary di-fucosylated and di-sialylated (*m/z* **4504.27**, **4953.49**) N-glycans were increased (Additional file S17). These core-fucosylated tetra-antennary N-glycans are associated with poor survival outcomes. Studies of cell lines suggested that core-fucosylation has a high impact on breast cancer cell metastasis related to the regulation of cell migration. It was also confirmed that core-fucosylated glycans were elevated in lymph node metastasis and tumors from patients with relapse^58^.

### Lymphovascular invasion

Lymphovascular invasion (LVI) is defined as the presence of tumor cells in a specific endothelial-lined space (lymphatics or blood vessels) surrounding the invasive carcinoma^59^. LVI is an important early prognostic factor in breast cancer, indicating a higher risk of the cancer spreading to other parts of the body, particularly the lymph nodes^60^.

Several research studies have consistently reported^61^ that breast cancers with LVI are associated with a higher risk of recurrence, and poorer overall survival rates, even when other factors such as tumor size and grade are taken into account. Patients with LVI may have tumor relapse after treatment, distant metastases, or reduced length of survival even though lymph nodes were negative.

Statistically significantly increased signals of N-glycans in tissues of patients with LVI were hybrid types or tri-antennary glycan structures (*m/z* **1810.93**, 1840.94, 1881.97, **2056.06**, 2086.07, bold *m/z* numbers mark core fucosylated glycans) when all subgroups were compared (Additional file S18). Interestingly, high mannose N-glycans (*m/*z 1350.69, 1799.91, 2004.01, 2208.11) and one hybrid glycan (*m/*z 2015.03) were increased only in NST samples with LVI (Additional file S18). NST tumor cells do not have unique histopathological features to distinguish them from the other types of breast cancer cells, but are decorated with the less-processed high mannose glycans in patients with LVI, in agreement with studies that suggested that high mannose N-glycans; may be associated with the development and progression of breast cancer^20,62^.

## Discussion

As complex N-glycans have often been associated with the metastatic potential of the cancer cells^46^, this finding may be surprising in the context that mucinous tumors tend to behave less aggressively than the more common subtypes^63^. On the other hand, the distinguishing structures could originate from secreted mucins, typical for this subtype, thus forming the majority of the tissue matter, as the name implies. Among other subtypes, the mean value of tri- and tetra- antennary N-glycans signals varied from 18.2% in L to 20.6% in HER2, with TNBC showing 19.8%. Control tissue samples showed a slightly lower representation at 18.0%. However, these differences were small compared to the expected biological variation in different individuals, with the standard deviation varying between 2 and 5%. Another aspect related to biological variation is the hypothesis that chronic social and economic stressors may be associated with an increased risk of cancer-associated N-glycan signatures in the tissue microenvironment in normal breast tissue^64^.

The effectiveness of any breast cancer treatment depends on the knowledge of tumor characteristics in terms of morphology, histological distinction, and biochemical indications. Since breast cancer is a heterogeneous disease, reliable subtyping and biochemical information on the tumor type can assist in selecting appropriate treatment and improving clinical outcomes. While extensive research has provided ample evidence that glycosylated proteins are involved in cancer progression and metastasis^20^, better biochemical insights are still needed to understand the complex repertoire of glycans in cancer-related processes.

Our study is informative in at least three different directions: (i) confirming that each histologically distinct breast cancer subtype has a quantitatively distinct set of N-glycans; (ii) enhancing biochemical information that could be utilized in future cancer treatment strategies; and (iii) establishing connection with clinical parameters. The comprehensive glycomic profiling methodology on a large patient cohort in this work emphasizes the occurrence of multi-antennary glycan structures in some cancer subtypes. Specifically, abnormally high levels of branched sialylated and fucosylated glycans were identified in mucinous cancer compared to other subtypes. A relatively lower abundance of multi-antennary N-glycans was found as a characteristic of the luminal subtypes. The finding concerning the enhanced level of high-mannose glycans in breast cancer tissues, when compared with normal tissues associated with breast cancer diagnosis was reported^65^ and can be connected with potential glycan-based targeted therapy or immunotherapy^66^ in the future. The elevation of high-mannose N-glycans was also correlated with vascular invasion and lymph node involvement^35^. Another alteration in the N-glycan profile described in our study involved bisecting glycans whose signal was enhanced in control tissues compared to breast cancer tissues. It correlates with the published results describing the overexpression of MGAT3 that significantly enhanced the bisecting N-GlcNAc on EGFR protein and suppressed the EGFR/Erk signaling, which further resulted in the reduction of migratory ability, cell proliferation, and clonal formation^53^.

Although studies dealing with glycomic subtyping are not numerous at this time, our data show agreement with several previously reported results. We also report data on N-glycan signatures relating to tumor size, proliferative rate, LVI, and metastases to lymph nodes, highlighting the importance of the often-overlooked paucimannosidic structures. While this comprehensive glycomic profiling technique has good clinical potential, further studies focusing on isomerism of sialic moieties could be crucial for understanding oncological consequences for different breast cancer cellular types. Recent research has shown that targeting N-glycan processing pathways may be a promising therapeutic approach for breast cancer^66–68^. In particular, inhibition of the enzyme N-acetylglucosaminyltransferase V (GnT-V) involved in the synthesis of certain N-glycans, has been shown to inhibit LVI and metastasis in breast cancer models^69^.

## Conclusions

Overall, while more research is needed to fully understand the relationship between N-glycans and breast cancer, current evidence suggests that aberrant N-glycosylation may be a promising target for the development of novel therapeutic approaches. In addition, there may be future potential for identifying specific cancer-associated N-glycans in the circulation for diagnosis, therapy decisions and patient monitoring. A related study on the representation of the N-glycans detected in this work is currently underway using sera collected from the same patient cohort.

### Supplementary Information

Supplementary Information 1.

Supplementary Information 2.

Supplementary Information 3.

Supplementary Information 4.

Supplementary Information 5.

Supplementary Information 6.

Supplementary Information 7.

Supplementary Information 8.

Supplementary Information 9.

Supplementary Information 10.

Supplementary Information 11.

Supplementary Information 12.

Supplementary Information 13.

Supplementary Information 14.

Supplementary Information 15.

Supplementary Information 16.

Supplementary Information 17.

Supplementary Information 18.

## Data Availability

The datasets used or analyzed in this study are available from the corresponding authors on reasonable request.

## Abbreviations

MALDI-TOF: Matrix-assisted laser desorption-ionization time-of-flight
MS: Mass spectrometry
ROTS: Reproducibility-optimized test statistic
CA 15-3: Cancer antigen 15-3
CEA: Carcinoembryonic antigen
IHC: Immunohistochemical
ER: Estrogen receptor
PR: Progesterone receptor
HER2: Human epidermal growth factor 2
Ki67: Marker of proliferation Ki-67
L: Luminal
LA: Luminal A
LB: Luminal B
LX: Luminal X
TNBC: Triple-negative breast cancer
EGFR: Epidermal growth factor receptor
AR: Androgen receptor
NST: No-specified-type
MUC: Mucinous carcinomas
ACN: Acetonitrile
TFA: Trifluoroacetic acid
DMF: N, N-dimethyl formamide
AUCn: Normalized peak area
FDR: False discovery rate
EMT: Epithelial-mesenchymal transition
LVI: Lymphovascular invasion
GnT-V: N-acetylglucosaminyltransferase V
