# Supplementary material for: N-glycan profiling of tissue samples to aid breast cancer subtyping
Source: Sci Rep. 2024 Jan 3;14:320. doi: 10.1038/s41598-023-51021-3 (PMC10764792; doi:10.1038/s41598-023-51021-3)
Supplement: Supplementary file 5 — Supplementary Information 5. [file 41598_2023_51021_MOESM5_ESM.pdf]

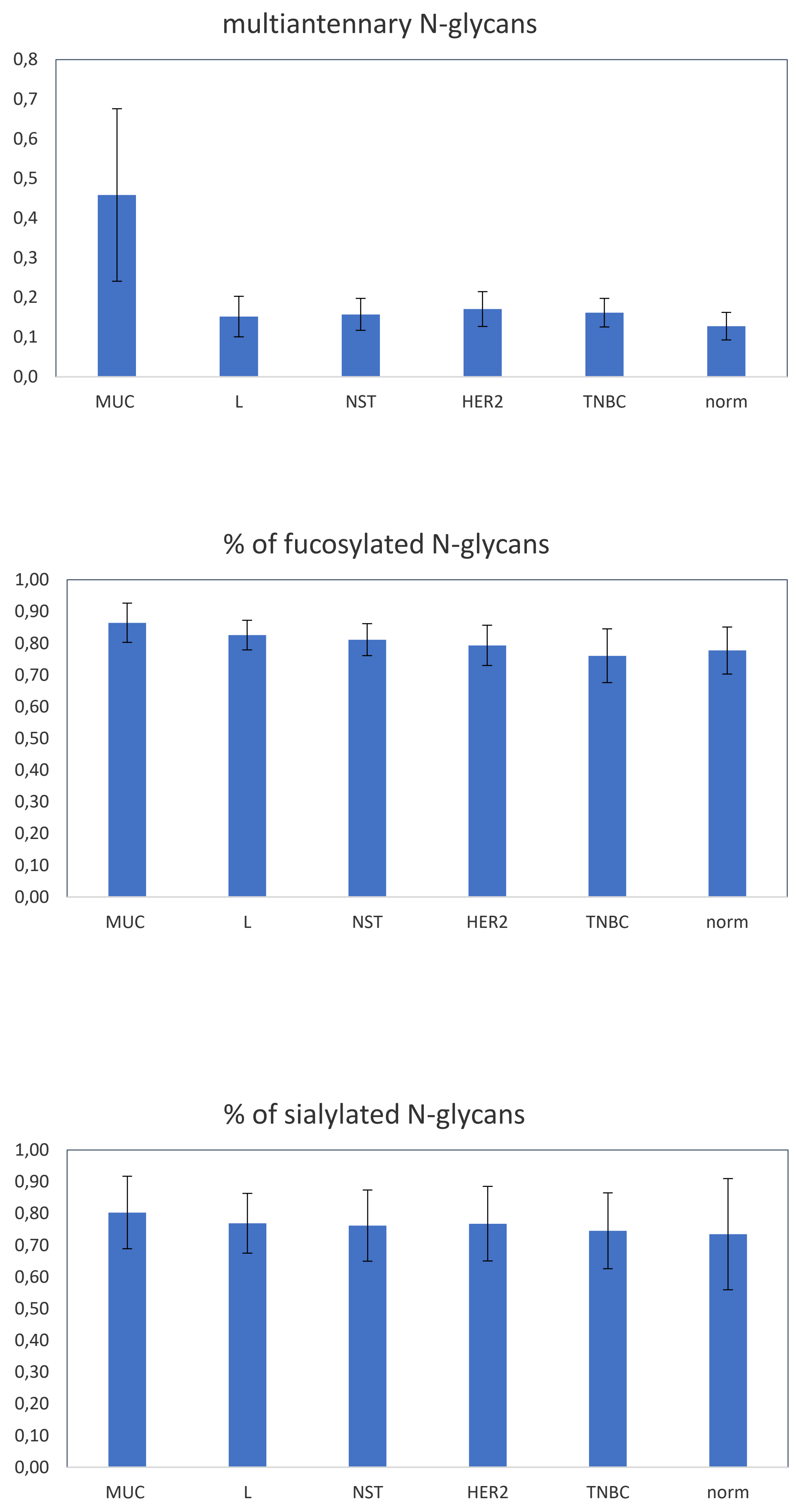

Changes in sialylated and fucosylated N-glycan structures.

Graphs show the percentage of multi-antennary N-glycans in each breast cancer subtype (top graph), fucosylated N-glycans (middle graph), and sialylated N-glycans (bottom graph), *y*-axes indicate percentages in decimal form.
