# Supplementary material for: N-glycan profiling of tissue samples to aid breast cancer subtyping
Source: Sci Rep. 2024 Jan 3;14:320. doi: 10.1038/s41598-023-51021-3 (PMC10764792; doi:10.1038/s41598-023-51021-3)
Supplement: Supplementary file 9 — Supplementary Information 9. [file 41598_2023_51021_MOESM9_ESM.pdf]

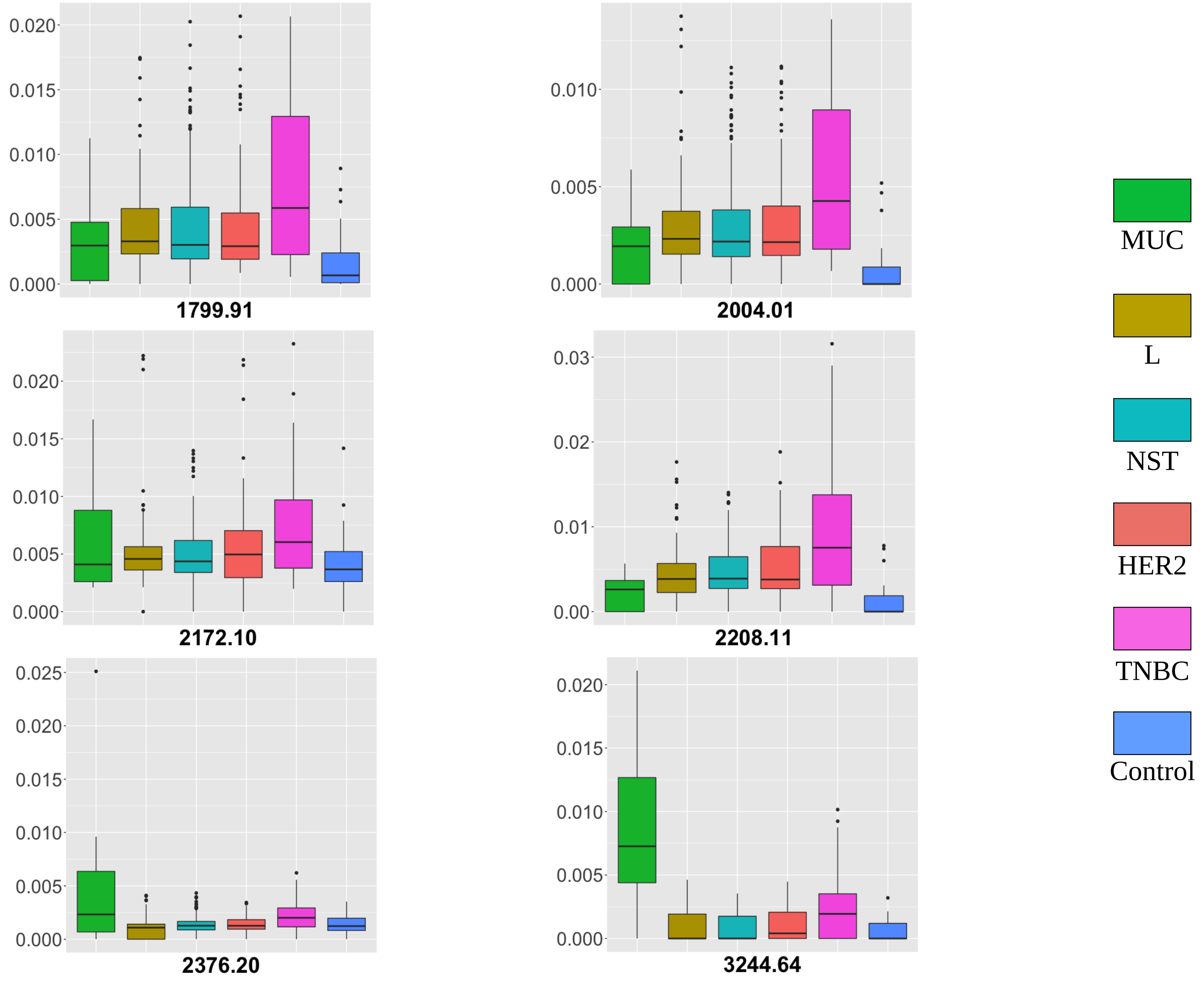

Changes of selected altered N-glycan signals in TNBC tissue samples compared to MUC, L, NST, HER2 subtypes, and controls. Box plots of normalized peak area (AUCn) of representative N-glycans in the subgroups and table with expanded information.
