# Supplementary material for: N-glycan profiling of tissue samples to aid breast cancer subtyping
Source: Sci Rep. 2024 Jan 3;14:320. doi: 10.1038/s41598-023-51021-3 (PMC10764792; doi:10.1038/s41598-023-51021-3)
Supplement: Supplementary file 11 — Supplementary Information 11. [file 41598_2023_51021_MOESM11_ESM.pdf]

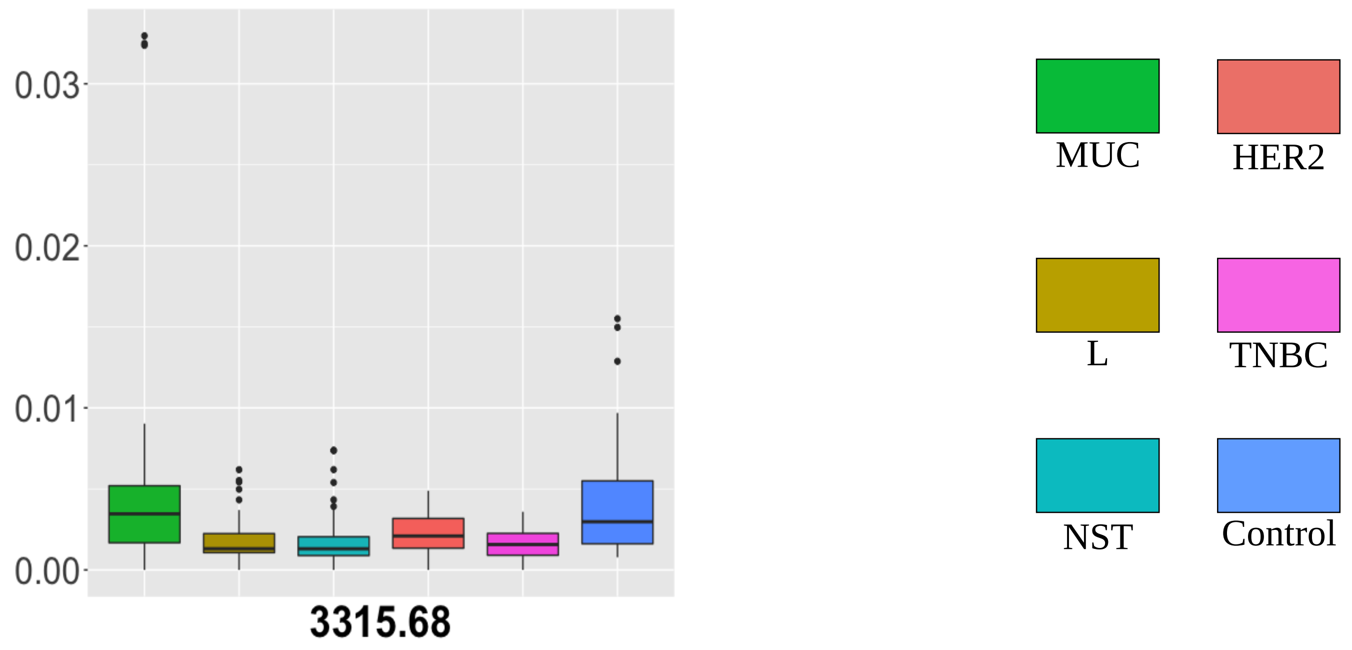

Changes of selected altered N-glycan signals in HER2 tissue samples compared to MUC, L, NST, TNBC subtypes, and controls. Box plots of normalized peak area (AUCn) of representative N-glycan in the subgroups and table with expanded information.
